# Effect of circadian rhythm, age, training and acute lameness on serum concentrations of cartilage oligomeric matrix protein (COMP) neo‐epitope in horses

**DOI:** 10.1111/evj.13082

**Published:** 2019-03-06

**Authors:** S. Ekman, A. Lindahl, U. Rüetschi, A. Jansson, K. Björkman, K. Abrahamsson‐Aurell, S. Björnsdóttir, M. Löfgren, L. Mattsson Hultén, E. Skiöldebrand

**Affiliations:** ^1^ Department of Biomedical Sciences and Veterinary Public Health Swedish University of Agricultural Sciences Uppsala Sweden; ^2^ Department of Clinical Chemistry and Transfusion Medicine Institute of Biomedicine Sahlgrenska University Hospital Gothenburg University Gothenburg Sweden; ^3^ Department of Anatomy, Physiology and Biochemistry Swedish University of Agricultural Sciences Uppsala Sweden; ^4^ Halland Animal Hospital Kungsbacka Horse Clinic Kungsbacka Sweden; ^5^ Agricultural University of Iceland Hvanneyri, Borgarnes, Saudarkrokur Iceland; ^6^ Department of Molecular and Clinical Medicine Wallenberg Laboratory Institute of Medicine The Sahlgrenska Academy, University of Gothenburg Gothenburg Sweden

**Keywords:** horse, COMP neo‐epitope, biomarker, lameness, serum, training, circadian

## Abstract

**Background:**

Molecular serum markers that can identify early reversible osteoarthritis (OA) in horses are lacking.

**Objectives:**

We studied serum concentrations of a novel cartilage oligomeric matrix protein (COMP) neo‐epitope in horses subjected to short‐term exercise and with acute lameness. The effects of circadian rhythm and age were also evaluated.

**Study design:**

Longitudinal studies in healthy horses and cross‐sectional comparison of lame and non‐lame horses.

**Methods:**

Sera were collected from five horses before and after short‐term interval exercise and during full‐day box rest. Sera from 32 acutely lame horses were used to evaluate age‐related effects. Independent samples from control horses (n = 41) and horses with acute lameness (n = 71) were included. COMP neo‐epitope concentrations were analysed using custom‐developed inhibition ELISAs validated for equine serum. The presence of COMP neo‐epitope was delineated in healthy and osteoarthritic articular cartilage with immunohistochemistry.

**Results:**

COMP neo‐epitope concentrations decreased after speed training but returned to baseline levels post‐exercise. No correlations between age and serum COMP neo‐epitope concentrations were found (r = 0.0013). The mean (±s.d.) serum concentration of COMP neo‐epitope in independent samples from non‐lame horses was 0.84 ± 0.38 μg/mL, and for lame horses was 5.24 ± 1.83 μg/mL (P<0.001). Antibodies against COMP neo‐epitope did not stain normal articular cartilage, but intracytoplasmic staining was found in superficial chondrocytes of mild OA cartilage and in the extracellular matrix of moderately osteoarthritic cartilage.

**Main limitations:**

ELISA was based on polyclonal antisera rather than a monoclonal antibody. There is a sex and breed bias within the groups of horses, also it could have been of value to include horses with septic arthritis and tendonitis and investigated joint differences.

**Conclusions:**

This COMP neo‐epitope can be measured in sera, and results indicate that it could be a biomarker for pathologic fragmentation of cartilage in connection with acute joint lameness.

## Introduction

Osteoarthritis (OA), a slow and progressive low‐grade inflammatory disease 1, 2, affects the athletic horse at an early age 3. Possible monitoring of early biochemical cartilage degradation is a prerequisite to alter progression from a reversible to an irreversible event 4. Imaging biomarkers are traditionally used to diagnose and study OA progression. However, different imaging techniques are limited in distinguishing normal tissue from early disease 5. This has shifted focus towards biochemical fluid (‘wet’) markers 4. Immunological assays are most commonly used to investigate OA in humans 6 and horses 7, 8. Specific antibodies that detect matrix components and proteinases in sera, urine and synovial fluids from horses have been used for enzyme‐linked immunosorbent assay (ELISA) 4. However, available biomarker assays are unable to distinguish pathologic fragmentation from normal cartilage turnover; instead, biomarkers of cartilage matrix changes must exclusively detect pathological degradation. Several studies 9, 10, 11 have shown that cartilage oligomeric matrix protein (COMP) in synovial fluid and serum is a promising biomarker of OA‐associated cartilage destruction in horses.

COMP, a major noncollagenous, 524‐kDa, homo‐pentameric protein, is present in articular cartilage where it binds collagen type II 12 and IX 13, cross‐bridging the collagen network. Synovial fluid COMP concentrations decrease in young horses during long‐term training and in OA joints 14. However, elevated concentrations were observed in equine joints with osteochondral fractures 15. These studies utilised ELISA with polyclonal antibodies that detect the native molecule and degraded monomers and further cleaved fragments. Recently 16, we analysed a unique COMP neo‐epitope in synovial fluids from horses and found that its concentration increased in animals with acute lameness compared to that in horses with chronic lameness and in non‐lame joints.

Our aim was to develop an ELISA that can measure this neo‐epitope in serum and delineate if short‐term training, circadian rhythm, age of the horse and acute lameness affect its concentration. We hypothesised that the neo‐epitope could be measured in serum, and that its concentration is not affected by sampling time, short‐term training or animal age. We also predicted that concentrations would increase with acute lameness similar to that observed for synovial fluid 16.

## Materials and methods

### Horses after short‐term exercise and box rest

Serum samples were collected from five Standardbred (STB) horses before and after a short‐term interval exercise session (Table 1). Five horses were also sampled several times during a 24‐h box rest 17, 18. The horses belonged to the Swedish Academy of Trotting and Thoroughbred Racing, Sweden. They were used for the education of apprentices and professional race trainers, and performed a simulated 2,140 m trotting race or interval training once a week. Lameness examination was not performed on these horses before exercise sessions or box rest. On days when no intensive exercise was performed, horses spent the morning hours in paddocks or were subjected to lighter exercise. The study took place between April and June 1995.

**Table 1:** Age, sex, breed and joint distribution of horses included in studies of a novel serum concentrations of cartilage oligomeric matrix protein (COMP) neo‐epitope

		Age	Sex	Breed					Joints				
Horses	N	Mean ± s.d. (min–max)	(n = M, G, S)	SWH	STB	Ponies	Icelandic horses	PRE	Coffin^a^	Fetlock^b^	Carpal	Tarsal	Stifle
Short‐term exercise study	5	7.0 ± 2.0 (5–10)	(0, 5, 0)	5					NA				
24 h box‐rest study	5	7.4 ± 1.9 (5–10)	(0, 5, 0)	5					NA				
Age correlation study	32	11 ± 4.7 (3–20)	(10, 21, 1)	21	2	8	1		7	28	13	9	0
Acutely lame horses	71	9.2 ± 4.6 (2–20)	(28, 34, 4)	35	20	11	3	2	15	45	52	9	2
Non‐lame horses	41	7.9 ± 4.6 (5–20)	(14, 0, 27)				41		NA				

N, number of horses; Min, minimum; Max, maximum; Sex: M, mare; G, gelding; S, stallion; NA, not applicable; SWH, Swedish Warmblood horses; STB, Standardbred horses; PRE, Pura Raza Española.

a Distal interphalangeal joint.

b Metacarpal/phalangeal/metatarsal/phalangeal joints.

#### Exercise tests

In the morning (08:00–12:00), exercise tests were performed on a field track. For this, the horses trotted five 500 m intervals at a speed of 11.1 ± 0.2 m/s (heart rate ~200 beats/min; heart‐rate recorder)^a^. After each interval, the horses returned to the start in a slow trot. This return trip took 2–4 min, depending on whether or not blood samples were collected. Exercise started with a warm‐up in slow trot (14 min). The horses returned to the stable in slow trot which took 13 min immediately after the last interval. An intravenous catheter was inserted into a jugular vein, in the evening before the exercise test, and flushed with heparinised saline (0.2% heparin) overnight and with isotonic saline between samplings. Blood samples were collected at rest 1), after warm‐up 2), before and after interval number four (time points 3 and 4), before and after interval number five (time points 5 and 6) and 15, 20, 30, 40 and 50 min after the last exercise interval (time points 7, 8, 9, 10 and 11). A final blood sample (time point 12) was collected before the next feeding the same day (1–4.5 h post‐exercise).

#### Circadian sampling

Five STB horses were used for blood sampling every hour for 24 h to determine the resting profile of each animal (Table 1). Additional blood samples were taken every 15 min for 1 h after the 17.30 and 05.30 meals were provided. Blood samples were taken via a catheter (Intranule [2.0 × 105 mm])^b^ introduced into one of the jugular veins. An extension tube was attached to the catheter to facilitate blood sampling. At 03:30, several horses were lying down; therefore, this sample was not collected.

### Horses of different ages

Blood samples were collected from 32 acutely lame horses of different ages that underwent a lameness examination at the University Animal Hospital (Uppsala, Sweden) (Table 1).

### Independent samples from control and lame horses

The inclusion criteria for the control horses (n = 14) recruited in the study were: considered to be sound by their owner, fit for breeding evaluation test including no radiological changes in their tarsal joints. Additional healthy stallions of the Icelandic horse breed (n = 27) with no radiological changes in their tarsal joints were included. These stallions were predicted to be fit for the riding test, included in a breeding evaluation, by their owners.

The inclusion criteria for the lame horses were: presented with acute lameness (duration not more than 2 weeks according to the owner) originating from one or more joints, and verified by a flexion test reaction, and that intra‐articular anaesthesia reduced the pain by up 75–100%. The horses were recruited from Kungsbacka Horse Clinic (n = 29), Gothenburg, Sweden and University Animal Hospital (n = 42), Uppsala, Sweden (Table 1).

All samples were stored in aliquots in −80°C to prevent unnecessary freezing and thawing.

### Neo‐epitope ELISA

The neo‐epitope at the N‐terminal part of one COMP fragment, containing five amino acids from the native COMP sequence (SGPTH), was detected in media at day 3 after interleukin‐1β stimulation in vitro 19.

An inhibition ELISA was developed to quantify the COMP neo‐epitope in serum, and was modified based on a previous protocol 16. In short, NUNC plates^c^ were coated with 4.0 μg/mL peptide (sequence, SGPTHEGVC) diluted in 0.1 mol/L carbonate buffer (pH 9.6), and incubated at 4°C overnight. A serial dilution of 5.0 μg/mL of peptide (sequence SGPTHEGVGMA) in 10 mmol/L PBS with 0.6% BSA and 0.8% SDS was used as the calibration curve (range = 5–0.078 μg/mL). Serum samples were diluted 1:10 in PBS with 0.89% SDS. Standard and serum samples were incubated in 96‐well Sterilin plates^c^ at 25°C overnight. On the second day, primary anti‐neo‐epitope of COMP antibody (diluted in PBS with 1% BSA and 4% triton‐X‐100) was added to the plates, which were incubated for 80 min at 25°C. NUNC plates were washed and blocked with 200 μL PBS with 1% BSA and 0.1% tween for 1 h, at 25°C. Next, 100 μL was transferred from the plates to NUNC plates and incubated for 1 h at 25°C. After incubation, NUNC plates were washed and secondary antibody (goat anti‐rabbit IgG H&L [HRP]) (Ab97051)^d^, diluted 1:20,000 in PBS with 1% BSA and 0.1% tween, was added. The plates were incubated for 1 h at 25°C and then washed six times and incubated with substrate (Substrate Reagent Pack DY999)^e^ for approximately 8 min at 25°C. Stop solution (1 mol/L H_2_SO_4_) was added, and the absorbance was measured at 450 nm (SpectraMaxPlus 384)^f^. The intra‐assay precision was determined by analysing 20 replicates of one sample with a medium concentration of the COMP neo‐peptide. The inter‐assay precision was determined through the analysis of 20 replicates from one sample on two occasions.

The detection limit for the assay was determined by analysing 20 replicate samples at varying concentrations.

Quality control samples consisting of pooled sera from 20 horses were run for every ELISA to ensure inter‐assay stability.

### Immunohistochemistry‐based cartilage sampling

Articular cartilage was from morphologically (macroscopically and microscopically) classified joints; one normal (carpal) sample and two with OA lesions (carpal and fetlock) were sampled at necropsy. Articular cartilage was immersed in 10% buffered formaldehyde, dehydrated, embedded in paraffin and tissue sections were stained with a polyclonal antibody against the COMP^g^ neo‐epitope (dilution 1:1000) and native COMP (Ab74524)^d^ (dilution 1:800) 16. Briefly, specimens were sectioned and mounted onto slides, deparaffinised, rehydrated and washed in phosphate‐buffered saline (PBS; 0.01 mol/L phosphate, 0.15 mol/L NaCl, pH 7.4). Endogenous peroxidase activity was quenched with 3% hydrogen peroxide in PBS. Nonspecific binding was blocked with 2% normal goat serum (X0907)^h^, prior to incubation with antiserum. After rinsing in PBS, sections were incubated with horseradish peroxidase (HRP)‐conjugated secondary antibodies (K5007) and visualisation (Real EnVision^™^ Detection System k5007 [ready‐to‐use kit])^h^ was performed using the colour developer 3, 3‐diaminobenzidine. For negative (isotype) controls, the primary antibody was substituted with nonimmune rabbit serum (X0936)^h^ at the same dilution used for the primary antibody. The sections were evaluated by light microscopy^i^.

### Data analysis

Values for COMP neo‐epitope in serum are presented as means and standard deviations (s.d.). Second‐degree polynomials in time points were fitted to data simultaneously for both short‐term exercise and box‐rested COMP values (series) with interaction terms for series and time, and series and squared times. A linear relationship between age and serum concentration of COMP neo‐epitope was investigated by calculating the correlation coefficient with a corresponding P value. This was performed parametrically (Pearson) and P≤0.05 was considered significant. The difference in means between lame and healthy horses was significantly different from 0 as tested by a two‐sample *t* test. The diagnostic performance of the ELISA that discriminated lame horses from healthy animals was evaluated by receiver operating characteristic (ROC) curve analysis 20. Statistical calculations were performed using SAS‐procedure mixed (SAS version 9.4)^j^ and ROC test was performed in GraphPad Prism (version 5.01 for Windows)^k^.

## Results

### ELISA

The ELISA for serum COMP neo‐epitope was determined to be valid with a detection limit of 0.156 μg/mL and inter‐assay variation of 10.6% and intra‐assay variation of 10.5%.

### Effects of short‐term exercise

**Figure 1:**
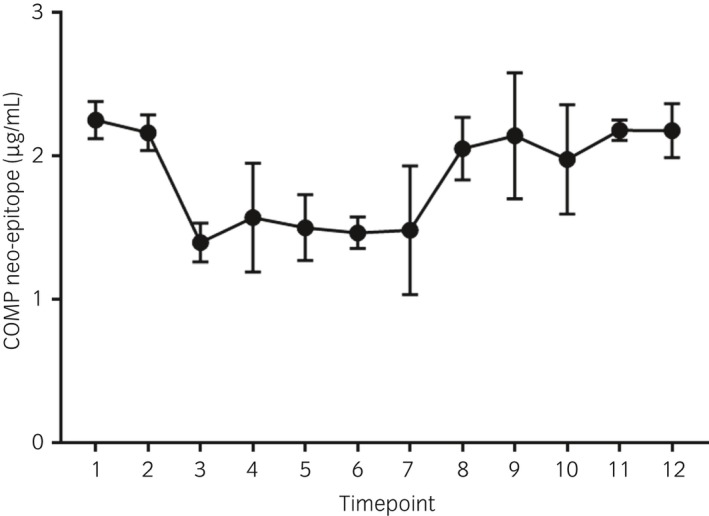
Mean (±s.d.) serum concentration of COMP neo‐epitope (μg/mL) in five horses after short‐term exercise. Blood samples were taken at rest 1), after warm‐up 2), before and after interval number four (time points 3 and 4), before and after interval number five (time points 5 and 6) and 15, 20, 30, 40 and 50 min after the last exercise interval (time points 7, 8, 9, 10 and 11). A final blood sample (time point 12) was collected before feeding the same day (1–4.5 h post‐exercise).

The mean (±s.d.) serum concentrations of COMP neo‐epitope at time points 1 and 12 were 2.25 μg/mL (±0.12) and 2.17 μg/mL (±0.19), respectively, during short‐term exercise (Fig 1). However, the curve was convex with an initial decrease that was followed by an increase up to the last time point; hence, the coefficient for squared time was positive and statistically significant. The equation for values for exercised horses was y = 2.5281−0.2467t + 0.01614t^2^, with P = 0.003 for time and P = 0.003 for time‐squared.

### Circadian effects

**Figure 2:**
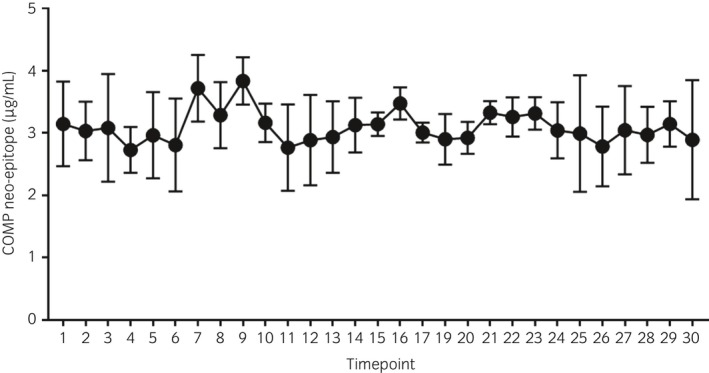
Mean (±s.d.) serum concentration of COMP neo‐epitope (μg/mL) in five horses during 24 h box rest. Blood samples were taken every hour for 24 h to determine the resting profile. Additional blood samples were taken every 15 min for 1 h after the 17:30 and 05:30 meals.

The mean (±s.d.) serum concentrations of COMP neo‐epitope at time points 1 and 30 (the last time point) were 3.14 (±0.68) μg/mL and 2.89 (±0.95) μg/mL respectively. The coefficients for time and time‐squared were not statistically significant; hence, no differences were identified during box rest (Fig 2).

The equation for values for box‐rested horses was y = 3.1143 + 0.002851t−4.79* 10^−6^ t^2^, with P = 0.9 for time and P>0.9 for squared times.

### Age‐related effects

**Figure 3:**
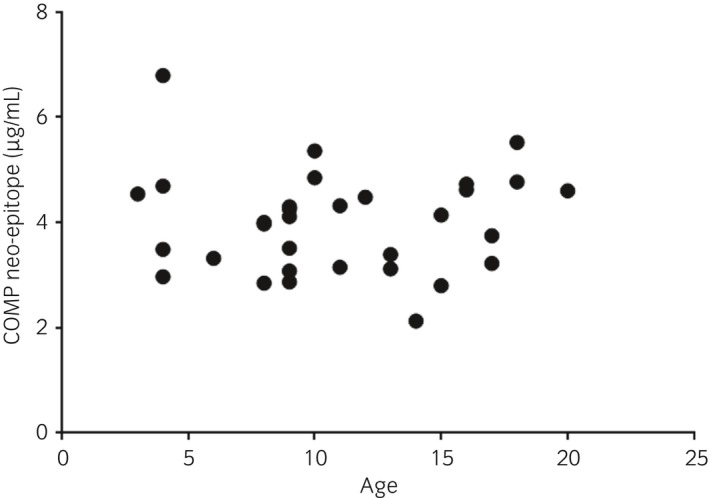
Serum concentrations of COMP neo‐epitope (μg/mL) related to age in 32 horses. The correlation coefficient for age and serum concentration was r = 0.0013 and the corresponding P value (0.99) indicated no correlation between these parameters.

The mean (±s.d.) age of the 32 horses was 11 (±4.7) years, with a range of 3–20 years, and the serum concentration of COMP neo‐epitope was 4 (±0.96) μg/mL. The correlation coefficient for age and serum concentration was r = 0.0013 and the corresponding P value (>0.9) indicated no correlation between these parameters (Fig 3).

### Independent samples from non‐lame horses and acutely lame horses

**Figure 4:**
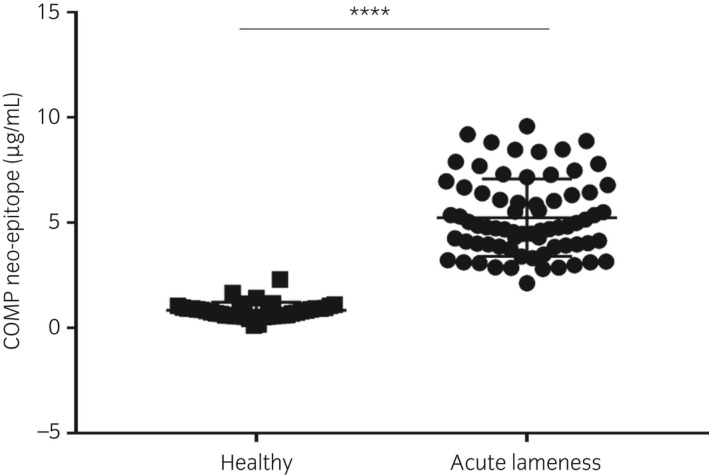
Serum concentration of COMP neo‐epitope (μg/mL) in independent samples from healthy horses (n = 41) and those with acute lameness (n = 71). Significantly increased COMP neo‐epitope concentrations were found in horses with acute lameness (mean ± s.d., 5.24 ± 1.83 μg/mL) compared to those in healthy horses (0.84 ± 0.38 μg/mL; P<0.001).

Forty‐one healthy horses, mean age (±s.d.) 7.9 ± 4.6 and 71 lame horses, age 9.2 ± 4.6, were included. The mean (±s.d.) serum concentrations of COMP neo‐epitope in independent samples were 0.84 ± 0.38 μg/mL and 5.24 ± 1.83 μg/mL for non‐lame and lame horses respectively (P<0.001; Fig 4). The area under the ROC curve was 0.99 (standard error, 0.0006) and the 95% confidence interval was 0.99–1.00, which was significantly different from 0.5 (P<0.0001).

### Immunohistochemistry analysis

**Figure 5:**
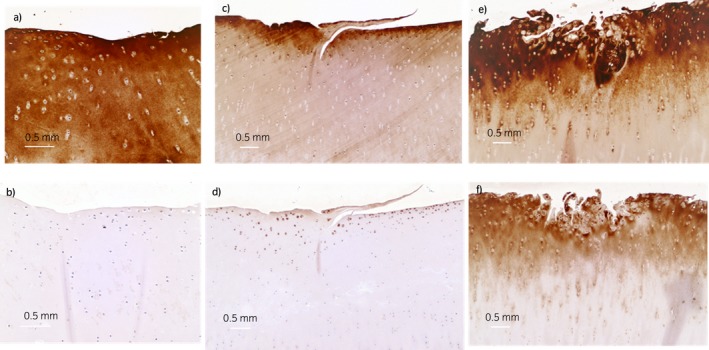
Immunohistochemistry analysis of articular cartilage of equine joints with normal articular cartilage a), b), articular cartilage with superficial mild fibrillation c), d) and that with fibrillation and cleft formation e), f), using a polyclonal antibody against the COMP neo‐epitope b), d), f) and a polyclonal antibody against native COMP (a, c, e). Scale bar = 0.5 mm.

The two OA joints were characterised as mild, with only focal superficial lesions of the articular cartilage, and moderate, with an area in the radial facet of cartilage fraying, cleft formations (superficial and middle layers) and erosions. The normal articular cartilage presented extracellular matrix (ECM) staining with antibodies against native COMP in all cartilage layers (Fig 5a); however, no COMP neo‐epitope staining (Fig 5b) was found. The articular cartilage with mild lesions showed staining with antibodies against native COMP in the ECM of all cartilage layers; however, fainter staining was observed in middle and deep layers compared to that in the superficial layer (Fig 5c). Antibodies against COMP neo‐epitope showed intracytoplasmic staining of the superficial chondrocytes in cluster formations (chondrones) surrounding small areas of acellular ECM (chondronecrosis) (Fig 5d). Adjacent single chondrocytes within the superficial zone displayed intracytoplasmic and ECM staining (Fig 5d).

Articular cartilage with moderate OA lesions showed intense staining in the ECM of all cartilage layers, with antibodies against native COMP (Fig 5e). Antibodies against the COMP neo‐epitope stained the chondrocytes in cluster formations and the ECM of superficial and middle articular cartilage layers (Fig 5f). The pericellular matrix surrounding the chondrones did not show immunostaining (Fig 5f). Controls did not result in staining in normal and OA articular cartilages.

## Discussion

Assays to detect OA joints have been developed that detect matrix components such as collagens, aggrecan and COMP and inflammatory cytokines in serum and synovial fluid. However, these biomarkers reflect both normal and pathologic matrix degradation and synthesis as well as overall inflammation. Available assays cannot detect early disease in individual animals, although they might indicate joint disease in populations. It is crucial that a biomarker can distinguish normal cartilage metabolism from pathologic fragmentation to identify joint pathology. Successful diagnostic assays will enable the detection of early OA and its progression on an individual level. Additionally, diagnosing acute lameness and monitoring treatment effects would be of great value. These assays can be used to adapt new physiological training programmes and reduce animal suffering.

This study was the first to show that increased serum concentrations of a unique COMP neo‐epitope are associated with acute lameness in horses. The ROC analysis assessing diagnostic and predictive accuracy showed that the test discriminates clearly between healthy and lame horses. In vivo results corresponded with a previous inflammatory explant model, in which the cleavage site of COMP increased after IL‐1β stimulation 19. Similar results were obtained for horses with chronic stages of OA, with increased concentrations of other COMP fragments in the synovial fluid, whereas no significant difference was found for serum levels of these COMP fragments in horses with OA compared to the levels in healthy horses 10, probably reflecting normal and pathologic cartilage turnover. Using sandwich ELISA based on a combination of unlabelled 2A11 and biotinylated 11 F10 monoclonal antibodies (mAbs), a significant increase in synovial fluid COMP concentrations was identified in horses with OA 10. However, mAbs that bound smaller COMP fragments did not show any change in serum and synovial concentrations in horses with experimentally induced osteochondral fractures; whereas, the concentration of COMP fragments was significantly higher in horses that developed osteophytes, suggesting increased COMP concentrations during severe chronic stages 11. In contrast, our study suggested that the unique COMP neo‐epitope (with N‐terminal cleavage site SGPTH), is a potential biomarker of acute lameness in individual horses.

Immunohistochemical staining of the COMP neo‐epitope in articular cartilage with mild and moderate destruction showed a different pattern, and no staining could be seen in normal articular cartilage. The cartilage with cleft formations had more prominent staining compared to that in the focally superficial fibrillated cartilage. The COMP neo‐epitope staining pattern in articular cartilage with lesions compatible with OA supports the higher serum concentrations found in acutely lame horses.

The STB horses used for circadian and short‐term exercise measurements were subjected to conventional race training. These horses were not clinically examined with flexion tests before entering the study. Hence, one can speculate that the higher serum concentrations (mean 2–3 μg/mL) compared to those in healthy horses (mean 0.84 μg/mL), might reflect horses with chronic joint pathology; however, they were not obviously lame upon sampling.

Our results show that serum COMP neo‐epitope concentrations are not influenced by time of day (circadian), short‐term exercise or age. However, there was a statistically significant decrease in concentrations after short‐term speed training 3, which could be explained by reduced plasma volumes 21 as one may speculate that the COMP fragment can leave the circulation due to the high hydrostatic pressure developed during high intensity exercise. However, after 20 min post‐exercise, values returned to baseline. Studies contradicting our results have been presented; serum concentrations of total and fragmented COMP in horses were found to decrease with age 22 and OA 23, and to increase after exercise 24 and in race horses with OA subjected to long‐term training (5 years) 25. Higher urine total COMP concentrations were also found in horses with septic and aseptic arthritis 26. Further, no changes in synovial fluid total COMP concentrations were found following arthroscopic removal of osteochondral fragments in horses 27. However, in contrast to the present study, these assays measure COMP fragmentation from normal turnover and pathologic destruction, explaining the reported different results. COMP measurements using different assays are not consistent; probably due to the use of antibodies that recognise internal/native epitopes and hence detect both intact and fragmented forms of the protein.

We chose Icelandic horses as control horses, since this breed is known to have OA in the distal tarsal joints (spavin) 28, but rarely in other joints 29. The horses included had normal radiographs of the tarsal joints and were clinically fit for the riding test. Serum concentrations of COMP neo‐epitope were low in these horses compared to those in horses with clinically acute lameness.

Other COMP neo‐epitopes have been identified in synovial fluid from patients with different joint diseases; patients with acute knee pain had the highest serum concentrations of these neo‐epitopes, as compared to those in patients with rheumatoid arthritis or OA 30. The cytokine IL‐1β has been shown to enhance the cleavage and release of COMP from tendon explants 31, with fragments being present in the early stage of tendon disease. The authors concluded that these fragments can ‘provide a platform for the development of neo‐epitope assays specific to injury stage for tendon disease’.

The COMP neo‐epitope quantified in the current study was also identified in an IL‐1β‐stimulated equine explant model 19 and was already degraded at day 3 of cytokine stimulation. Its concentration was found to increase in synovial fluid 16 and sera from horses presenting with acute lameness. Furthermore, immunolabelling detected the COMP neo‐epitope in the cytoplasm of chondrocytes surrounding the fibrillation of the mild and severe OA articular cartilage, but not in chondrocytes of normal articular cartilage. Its presence in superficial and deep fraying of articular cartilage and the increase in serum and synovial concentrations 16 in horses with acute lameness suggest that this neo‐epitope has a role in the early and acute stages of equine OA. Hence, unique COMP fragmentation related to cytokine stimulation could be valuable for monitoring the stages of connective tissue damage.

Our results also show that short‐term training does not influence serum concentrations of this unique COMP neo‐epitope. This is in accordance with results in humans, wherein no changes in serum COMP concentrations were observed after 1 h of running 32 or during a follow‐up of long‐term training in volleyball athletes 33.

Easy‐to‐use biomarker assays that can diagnose the early painful OA stage would be of great value, especially in young racehorses. If early joint disease is identified, it will be possible to change the training programme and treat the joint inflammation, which could prevent OA progression.

In conclusion, the present study showed that serum concentrations of a unique COMP neo‐epitope in horses represent a potential biomarker for acute joint disease that is not affected by age, short‐term training or circadian rhythms during rest. The production of monoclonal antibodies has been initiated to establish a sandwich ELISA. Further studies must be performed using a larger population of healthy and diseased horses, and these reference values will provide the basis for developing a diagnostic tool. Additional to future studies we would investigate whether different joints can influence the levels.

## Authors’ declaration of interests

No competing interests have been declared.

## Ethical animal research

The Ethical Committee on Animal Experiments, Stockholm, Sweden approved the study protocol (Dnr; N378/12).

## Owner informed consent

Horse owners provided informed consent for the collection of serum for biobanking for research purposes.

## Sources of funding

Western Region Research Funding (ALF GBG‐716171), the Swedish‐Norwegian Foundation for Equine Research (H0947014), Swedish Research Council grant (VR 2015‐02959) and the Swedish Research Council for Environment, Agricultural Sciences and Spatial Planning (FORMAS 221‐2013‐317) supported this research.

## Authorship

S. Ekman contributed to study design, data analysis and interpretation and preparation of the manuscript. A. Lindahl contributed to study execution, data analysis and interpretation and preparation of the manuscript. U. Rüetschi, K. Björkman and L. Mattson Hultén contributed to study design and study execution. A. Jansson contributed to study execution and preparation of the manuscript. K. Abrahamsson‐Aurell, S. Björnsdóttir and M. Löfgren contributed to study execution and data analysis and interpretation. E. Skiöldebrand contributed to study design, study execution, data analysis and interpretation and preparation of the manuscript. All authors gave their final approval of the manuscript.

## Manufacturers' addresses

^a^ Optipuls, Borlänge, Sweden.

^b^SweVet‐Piab, Sjöbo, Sweden.

^c^Thermo Fisher Scientific, Waltham, Massachusetts, USA.

^d^Abcam, Cambridge, UK.

^e^R&D Systems, Abingdon, Oxford, UK.

^f^MDS Analytical Technologies Ltd, Wokingham, Berkshire, UK.

^g^Genscript, Piscataway, New Jersey, USA.

^h^DAKO Sweden AB, Stockholm, Sweden.

^i^Nikon Instruments Inc., Melville, New York, USA.

^j^SAS Institute Inc, Cary, North Carolina, USA.

^k^GraphPad Software, La Jolla, California, USA.
